# *In Vitro* and *in Vivo* Anticancer Activity of Pardaxin against Proliferation and Growth of Oral Squamous Cell Carcinoma

**DOI:** 10.3390/md14010002

**Published:** 2015-12-23

**Authors:** Yifan Han, Zhibin Cui, Yen-Hsing Li, Wei-Hsuan Hsu, Bao-Hong Lee

**Affiliations:** 1 Department of Oral Pathology, Ninth People’s Hospital, Shanghai Jiao Tong University School of Medicine, Shanghai 200000, China; yifanhan2014@gmail.com; 2 Department of Comparative Pathobiology, Purdue University, West Lafayette, IN 47907, USA; cuizhibin1985@gmail.com; 3 Department of Chemistry, Purdue University, West Lafayette, IN 47907, USA; li4094@gmail.com; 4 Biochemical Process Technology Department, Center of Excellence for Drug Development, Biomedical Technology and Device Research Laboratories, Industrial Technology Research Institute, Rm. 103, Bldg. 27, 321, Sec. 2, Kuang Fu Rd., Hsinchu 100401, Taiwan; 5 Division of Hematology and Oncology, Department of Internal Medicine, Taipei Medical University Hospital, Taipei 110, Taiwan; 6 Department of Traditional Chinese Medicine, Taipei Medical University Hospital, No.252, Wu Hsing Street, Taipei 110, Taiwan

**Keywords:** pardaxin, antimicrobial peptide (AMP), oral squamous cell carcinoma (OSCC) cells, 7,12-dimethylbenz[a]anthracene (DMBA), oral cancer

## Abstract

Pardaxin (H-GFFALIPKIISSPLFKTLLSAVGSALSSSGGQE-OH), a 33-amino-acid polypeptide, is an antimicrobial peptide (AMP) isolated from the marine fish species *Pardachirus marmoratus*. Pardaxin shows antibacterial and antitumor activities. However, pardaxin-induced inhibition of oral cancer and the mechanism of tumor reduction in buccal pouch carcinogenesis after pardaxin painting remain undetermined. Additionally, the toxic effects of pardaxin on normal tissue remain unclear. The present study investigated the anticancer activity of pardaxin in oral squamous cell carcinoma (OSCC) cells in the hamster buccal pouch model with or without 7,12-dimethylbenz[a]anthracene (DMBA) pretreatment. This is the first study to confirm the effects of pardaxin on normal tissue and its nontoxic effects *in vivo*. Cell viability assays and colony formation tests in OSCC cell lines (SCC-4) demonstrated that pardaxin reduced cell viability in a dose-dependent manner. Immunofluorescence staining of cleaved caspase-3 in SCC-4 cells revealed that expression of activated caspase-3 in SCC-4 cells significantly increased after 24-h treatment with pardaxin. Additionally, a cell cycle analysis indicated that pardaxin treatment resulted in the cell cycle arrest of SCC-4 cells in the G2/M phase, thereby limiting cell proliferation. Furthermore, pardaxin treatment substantially alleviated carcinogenesis in the DMBA-induced hamster buccal pouch model by lowering prostaglandin E_2_ levels. These results suggest that pardaxin is a potential marine drug for adjuvant chemotherapy for human OSCC and oral cancer.

## 1. Introduction

Oral cancer is one of the most common cancers worldwide and responsible for 135,000 deaths annually [1]. Oral squamous cell carcinoma (OSCC) accounts for 90% of oral malignant tumors. In Taiwan, oral cancer is the fourth most common cancer and was the fifth leading cause of death in Taiwanese males in 2012 [2]. To improve patient survival and the quality of life, new therapeutic approaches focusing on molecular targets and mechanisms that mediate tumor cell growth or cell death have gained attention. Cancer therapeutic targets, such as gain-of-function mutations in oncogenes and loss-of-function mutations in tumor suppressor genes, are the key factors for carcinogenesis in the head and neck. Furthermore, the current view of a tumor as an organ that can communicate with its surrounding stroma indicates that the tumor microenvironment plays a vital role in tumor progression and provides a new perspective for cancer prevention and therapy using inflammatory mediators such as prostaglandin E_2_ (PGE_2_) [1,3].

Antimicrobial peptides (AMPs) belong to a large family of peptide molecules that typically contain <100 amino acids. They exist in various types of cells in both vertebrates and invertebrates. Previous studies have reported that AMPs facilitate promoting human health and reduce the cancer risk [4]. Moreover, AMPs play a crucial role in regulating the innate system, angiogenesis, and anticancer processes [5,6,7]. Furthermore, AMPs can specifically target certain proteins on the cancer cell membrane and induce cancer cell death, thus exhibiting potent toxicity in targeted cancer cells. Therefore, they have potential for application in antitumor therapy [8,9,10,11]. The present study investigated the anticancer role of pardaxin, an AMP, in OSCC cell lines and a hamster model along with its potential molecular mechanism.

Pardaxin can inhibit various cancer cells and tumors, including canine perianal gland adenomas [12], bladder-associated tumors [13], human fibrosarcoma cells [7], and murine fibrosarcoma cells [14]. However, pardaxin-induced cancer cell inhibition in oral cancer remains unclear. The mechanism of tumor reduction in buccal pouch carcinogenesis after pardaxin painting remains unclear. The present study investigated the anticancer role of pardaxin in OSCC cells and in the 7,12-dimethylbenz[a]anthracene (DMBA)-induced hamster buccal pouch model.

## 2. Results

### 2.1. Pardaxin-Induced Inhibition of Cell Growth in SCC-4 Cells

**Figure 1:**
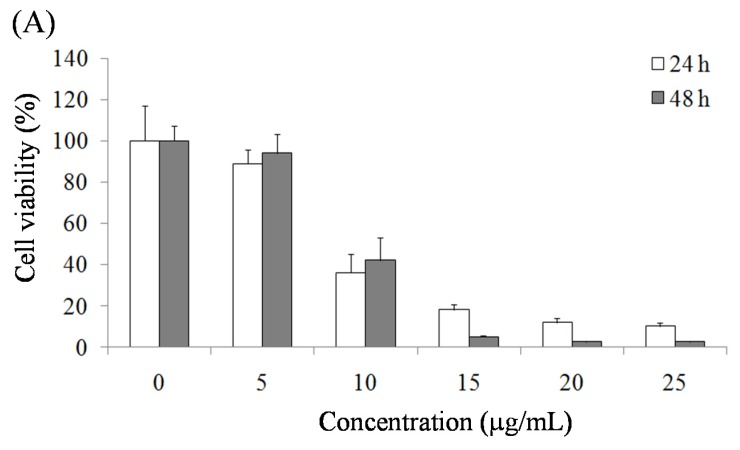

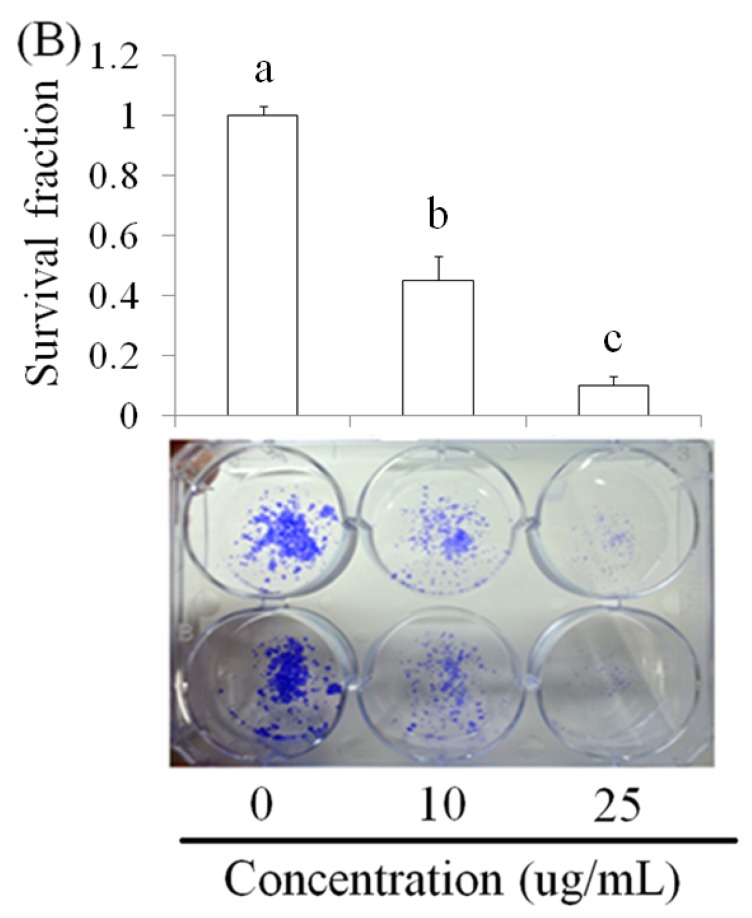
(**A**) Inhibitory effects of pardaxin on viability of SCC-4 cells after 24-h and 48-h treatment according to the MTT assay. (**B**) Suppressive effects of pardaxin on proliferation of SCC-4 cells after seven-day treatment according to crystal violet staining. Data are presented as the mean ± SD (*n* = 3). Significant differences are shown using different letters (*p* < 0.05).

The effect of pardaxin on the viability of SCC-4 cells was assessed using an MTT assay to measure the mitochondrial activity of living cells. The results demonstrated that pardaxin suppressed the growth rate of SCC-4 cells in a dose-dependent manner (0, 5, 10, 15, 20, and 25 μg/mL) (Figure 1A). Moreover, a colony formation assay revealed that the number of SCC4 cell colonies substantially decreased on the seventh day after treatment with pardaxin at the concentrations of 10 and 25 μg/mL (Figure 1B). The inhibition of SCC-4 cell growth by pardaxin suggested suppression of OSCC cells.

### 2.2. Pardaxin-Induced Apoptosis in OSCC Cells

**Figure 2:**
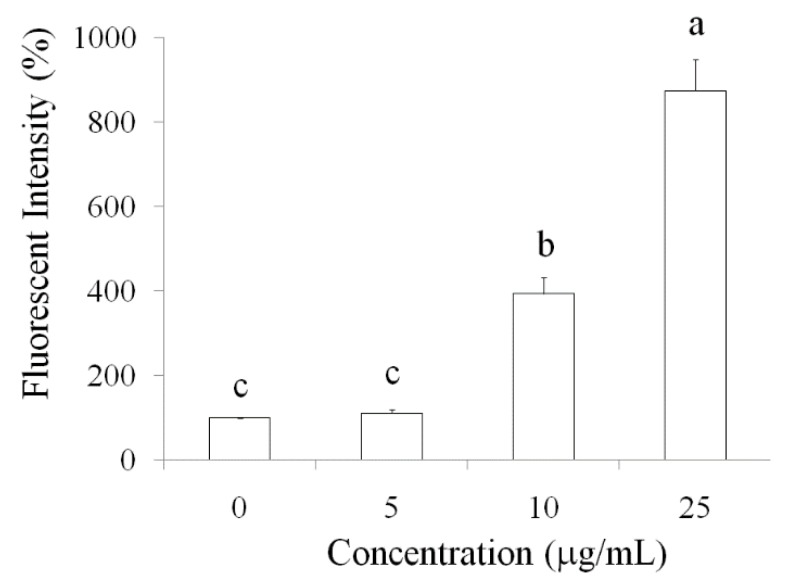

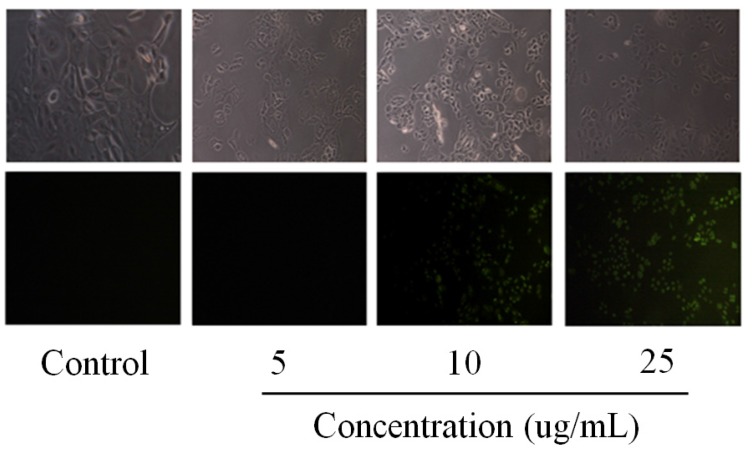
Measurement of caspase-3 expression by fluorescent intensity in SCC-4 cells after various concentrations of pardaxin (5, 10, and 25 μg/mL) treatment for 24-h observed using fluorescent microscopy.

The potential of pardaxin induced apoptosis in various cancer cells has been reported [7,12,13,14], but it remains unclear in pardaxin-treated oral cancer cells inhibition. Caspase-3 is important molecule in downstream of apoptotic pathway. Therefore, we investigated the elevation of caspase-3 expression by pardaxin treatment in SCC-4 cells. Caspases are essential for apoptosis and cell development. We investigated SCC-4 cells treated with various concentrations (0, 5, 10, 25 μg/mL) of pardaxin by using immunofluorescence staining of cleaved caspase-3 after 24 h. The results revealed that the fluorescence of cleaved caspase-3 was enhanced after pardaxin treatment (Figure 2). These results suggested that activation of caspase-3 was induced by pardaxin and subsequently led to apoptosis in OSCC cells.

### 2.3. Cell Cycle of OSCC Cells after Treatment with Pardaxin

**Figure 3:**
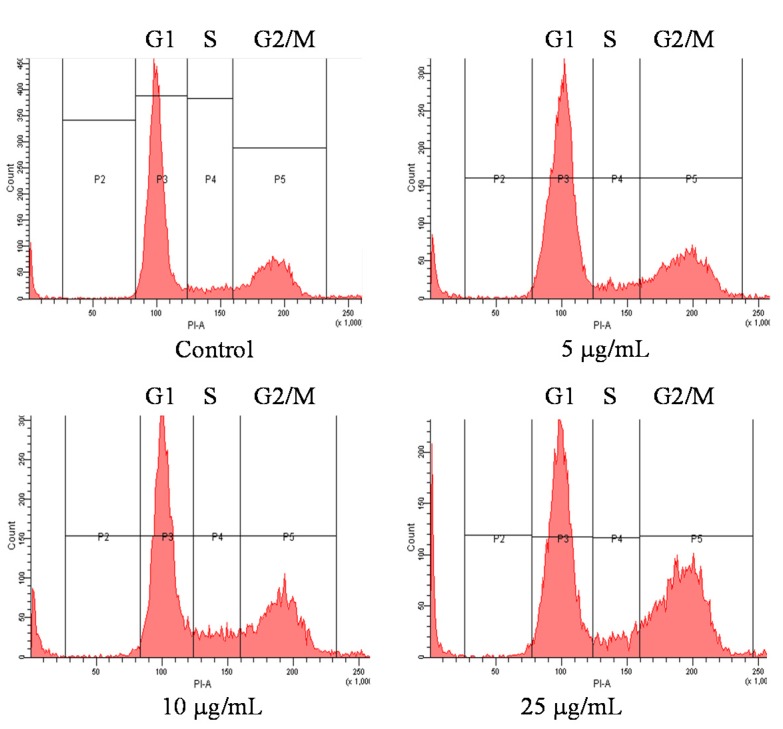
Cell cycle arrest of SCC-4 cells after 12-h pardaxin treatment measured using flowcytometry. Data are presented as the mean ± SD (*n* = 3). Significant differences are shown using different letters between a and b (*p* < 0.05).

The inhibition of cell viability can result from the induction of apoptosis or cell growth suppression. Therefore, to clarify the cellular processes possibly affected by pardaxin, its effect on the cell cycle was investigated using flow cytometry and propidium iodide (PI) staining. The results revealed that pardaxin treatment for 12 h at three concentrations (5, 10, and 25 μg/mL) profoundly affected the cell cycle of SCC-4 cells. Pardaxin induced a reduction of the cell population in the G0/G1 phase and counterbalanced this with the cell arrest in the G2/M phase (Figure 3).

**Table:** 

	G0/G1	S	G2/M
Untreated	73.6 ± 5.22 ^a^	6.6 ± 0.15 ^b^	19.8 ± 2.05 ^c^
5 μg/mL	67.7 ± 4.98 ^a^	5.9 ± 0.14 ^b^	26.4 ± 1.73 ^b^
10 μg/mL	59.0 ± 4.96 ^b^	10.2 ± 0.36 ^a^	30.8 ± 3.91 ^b^
25 μg/mL	54.4 ± 4.11 ^b^	8.6 ± 0.08 ^a^	37.0 ± 3.24 ^a^

**Figure 4:**
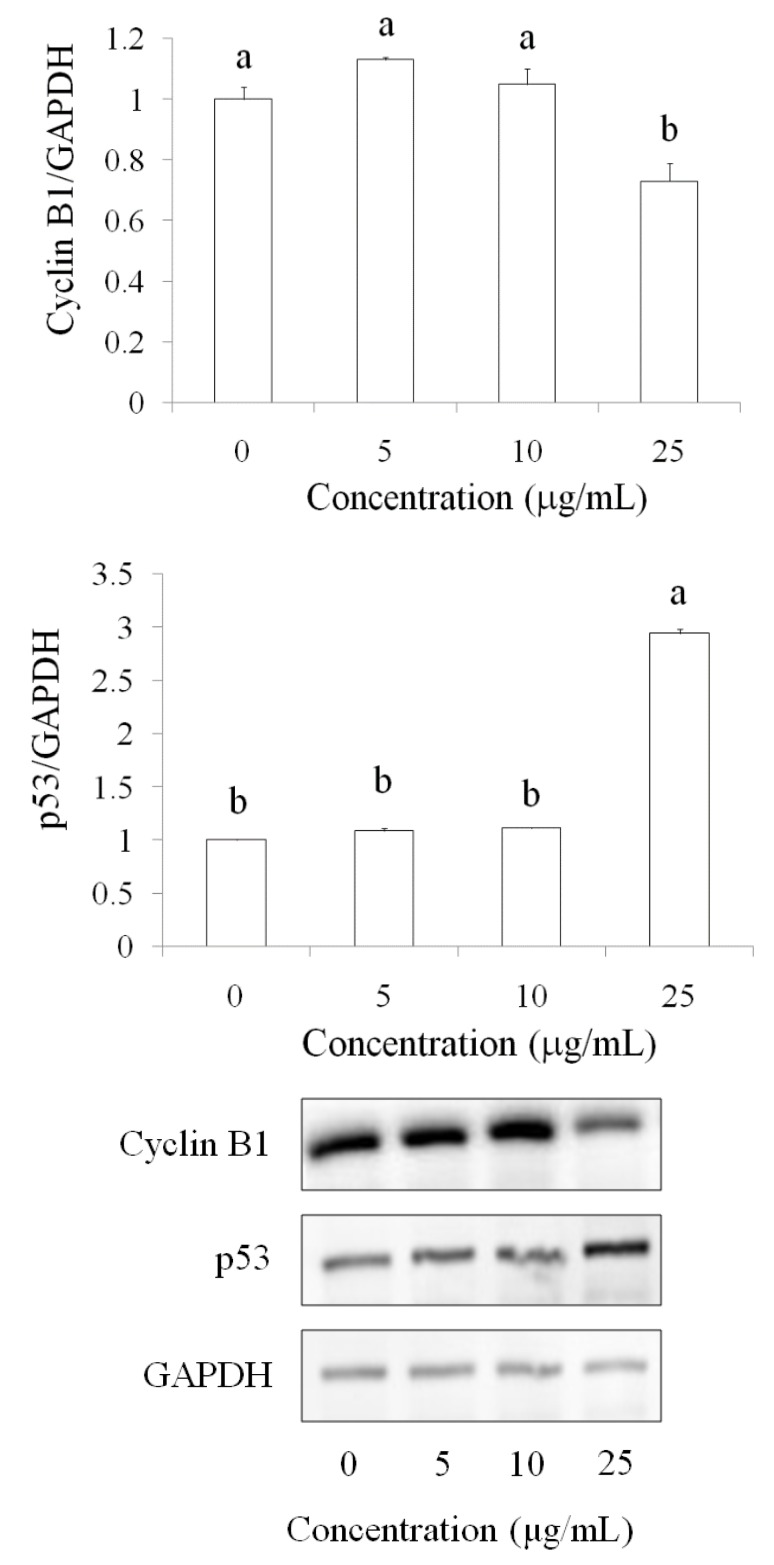
Regulation of cyclin B1 and p53 after pardaxin treatmentfor 24 h in SCC-4 cells. Cyclin B1 and p53 protein levels were detected using the Western blot technique. Data are presented as the mean ± SD (*n* = 3). Significant differencesare shown using different letters between a and b (*p* < 0.05).

Western blot results demonstrated that the cyclin B1 protein level decreased after pardaxin treatment for 24 h in a dose-dependent manner (5, 10, and 25 μg/mL), whereas the p53 protein level was upregulated by pardaxin (Figure 4). These results suggested that pardaxin inhibited SCC-4 cell growth and led to G0/G1 phase reduction and G2/M arrest, probably because of the regulation of cyclin B1 and p53 by pardaxin.

### 2.4. Pardaxin-Induced Reduction in Carcinogenesis in DMBA-Induced Hamster Buccal Pouch Model

**Figure 5:**
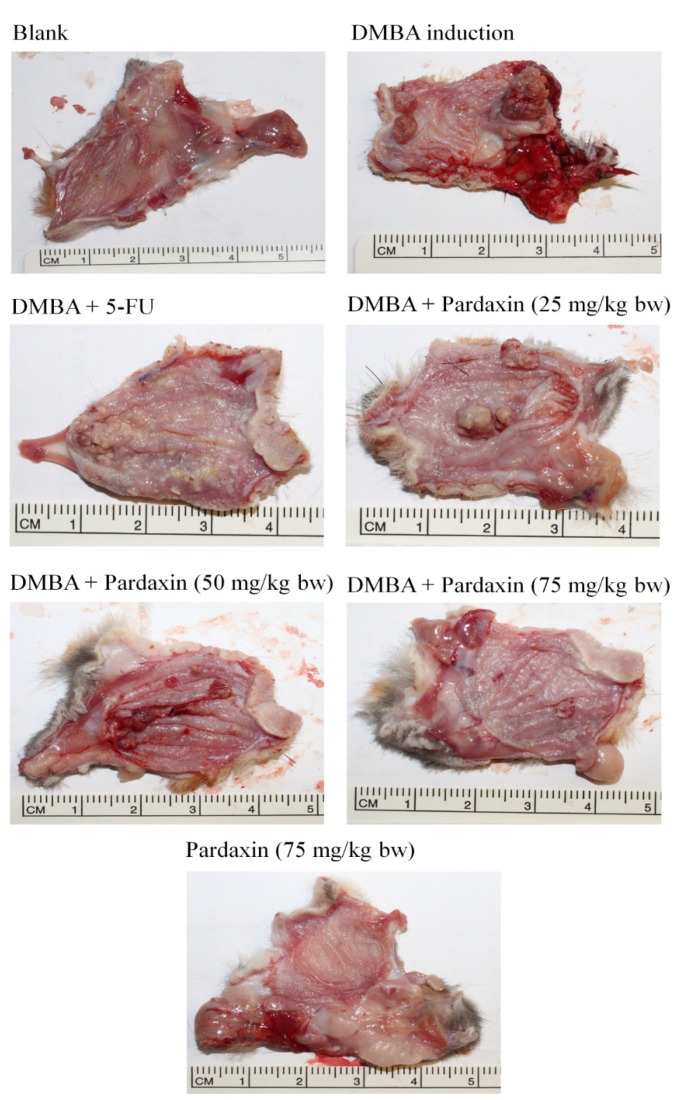
Pardaxin-reduced carcinogenesis in the DMBA-induced hamster buccal pouch model (*n*= 4–6). Tumorigenesis was monitored using DMBA alone or DMBA with different doses of pardaxin and 5-FU.DMBA: 7,12-dimethyl-1,2-benz[a]anthracene. 5-FU: 5-fluorouracil.

To investigate the potential application of pardaxin therapy in OSCC, the DMBA-induced hamster buccal pouch model was used. In the control group, in which hamsters were treated with mineral oil only, no tumor formation was observed. After eight-week pretreatment with DMBA, a tumor formed in the hamster buccal pouch. Pardaxin treatment (25–75 mg/kg bw) for six weeks after DMBA pretreatment significantly lowered the mean tumor volume and substantially reduced the tumor burden in a dose-dependent manner (Figure 5 and Table 1).

**Table 1:** Pardaxin-induced inhibition of oral tumor in DMBA-induced hamster buccal pouch model.

Group	Mean Tumor Volume (mm^3^)	Tumor Burden (mm^3^)	Mean Tumor Number/Hamster
Control	0	0	0
DMBA	131.4	417.6	13.5
DMBA + Pardaxin (25 mg/kg bw)	138.9	398.1	12.9
DMBA + Pardaxin (50 mg/kg bw)	89.3	237.7	9.1
DMBA + Pardaxin (75 mg/kg bw)	36.2	74.8	5.1
DMBA + 5-FU (25 mg/kg bw)	41.5	95.4	4.8
Pardaxin (75 mg/kg bw)	0	0	0

Data are presented as the mean ± SD (*n* = 4–6).

### 2.5. Inhibition of PGE_2_Levels in DMBA-Induced Hamster Buccal Pouch Tumor Model by Pardaxin

**Figure 6:**
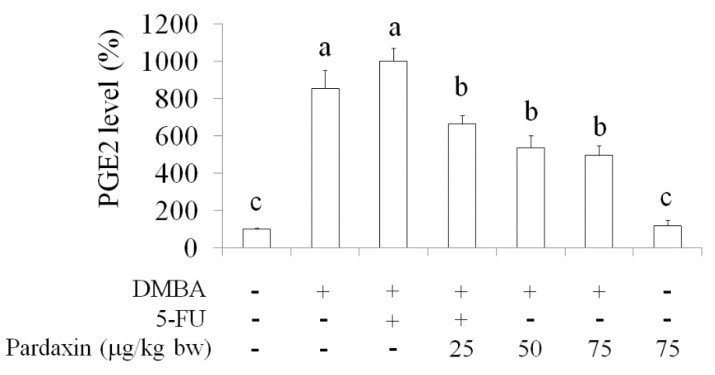
Pardaxin-induced suppression of serum PGE_2_ levels in the DMBA-induced hamster buccal pouch model. Data are presented as the mean ± SD (*n* = 4–6). Significant differences are presented using different letters, a, b and c (*p* < 0.05). DMBA: 7,12-dimethyl-1,2-benz[a]anthracene. 5-FU: 5-fluorouracil.

5-Fluorouracil (5-FU) is one of the most effective agents used in oral cancer treatment [15,16]. 5-FU is widely used in clinical cancer therapy, particularly in head and neck cancers. The effect of pardaxin on PGE_2_, a proinflammatory lipid mediator, increased to a high level in a mouse tumor model and hamster buccal pouch model after different treatments (Figure 6). Compared with mice treated with DMBA alone, the serum level of PGE_2_ was reduced in the group treated with pardaxin (75 mg/kg bw). The suppression effect of PGE_2_ by pardaxin (75 mg/kg bw) was effectively lowered in DMBA-induced hamster; in contrast, 5-FU treatment significantly increase PGE_2_ as DMBA induction. Moreover, pardaxin (75 mg/kg bw) did not result in elevation of PGE_2_in hamster without DMBA induction. These results suggested that pardaxin lowered the levels of PGE_2_, a tumor progression contributor, and may regulate COX signaling in OSCC *in vivo* as well as safe potential.

## 3. Discussion

OSCC has the fastest rate of growth in incidence and highest mortality rate of all cancers and is the sixth most common cause of cancer death in Taiwan. However, the underlying mechanisms of oral cancer development have not been entirely elucidated [1]. Understanding the molecular mechanisms involved in the progression of oral cancer facilitates developing new therapeutic strategies. Several AMPs have demonstrated potential as targeted therapeutic anticancer agents in various cancer types [17,18]. AMPs induce cancer cell apoptosis and cell cycle arrest, thus proving their toxicity toward cancer cells [18,19,20,21]. The present study investigated the anticancer effects of pardaxin in OSCC cells and animal models.

Several studies have reported that AMPs can induce apoptosis in cancer cells. In HeLa cells, pardaxin was observed to trigger activator protein-1 and induce programmed cell death [22]. Furthermore, it inhibited tumor growth factor-beta generation in HeLa cells [23]. In the present study, pardaxin inhibited SCC-4 cell growth and induced apoptosis in SCC-4 cells through upregulation of caspase-3 activity (Figure 2). Caspases play a vital role in both the initiation and execution of apoptosis and are essential for cellular DNA fragmentation [24]. There are two types of caspase proteins, initiator caspases and effector caspases. Caspase-3 is an effector caspase [24]. It cleaves other protein substrates within the cell, resulting in apoptosis. The p53 protein has been intensively studied as a major tumor suppressor that detects oncogenic events in cancer cells [25]. Our results demonstrated that caspase-3 accumulated in cells treated with pardaxin, and its expression increased with pardaxin treatment in a dose-dependent manner. This suggested that pardaxin induces cell apoptosis through caspase signaling and leads to cancer cell death. Furthermore, the cell cycle analysis experimental results revealed that pardaxin induced G2/M phase arrest during cell growth, probably by inhibiting cyclin B1 expression and promoting p53 expression. Our experimental results demonstrated that cyclin B1 expression decreased after treatment with an increased concentration of pardaxin. By contrast, p53 expression increased apparently (Figure 3 and Figure 4). These results collectively demonstrated that pardaxin lowered cyclin B1 expression and upregulated the p53 protein level, leading to G2/M phase arrest and cell proliferation inhibition in OSCC cells. The mechanism of pardaxin-mediated regulation of apoptotic and cell cycle-related genes requires further investigation.

PGE_2_, a proinflammatory mediator, is involved in chronic inflammation that facilitates cancer progression, growth, and proliferation [26,27]. PGE_2_ facilitates the acquisition of malignant hallmarks by cancers [28]. Serum PGE_2_ levels increased in both mouse tumor models and several cancer patients, including those with head and neck cancer [29,30]. Furthermore, the COX signaling pathway plays an essential role and PGE_2_ levels are elevated in oral cancer [31,32]. Our results suggested that pardaxin can attenuate PGE_2_ generation caused by DMBA induction in a dose-dependent manner (Figure 6). Because the elevated PGE_2_ levels were higher after 5-FU treatment than after DMBA induction, 5-FU might have exerted greater cytotoxicity in oral tissue. In addition, a high pardaxin level did not result in oral tissue damage in hamsters (Figure 5 and Table 1). Our study revealed that 75 mg/kg bw of pardaxin exhibited the same efficacy as that of 5-FU in mice with oral cancer, suggesting that natural pardaxin is a promising agent for use in OSCC chemotherapy.

## 4. Experimental Section

### 4.1. Cell Culture

Human SCC-4 cell lines were obtained from the Bioresource Collection and Research Center (Hsinchu, Taiwan). SCC-4 cells were cultured in the DMEM/F12 medium (GIBCO BRL; Thermo, Carlsbad, CA, USA), supplemented with 10% heat-inactivated fetal bovine serum (GIBCO BRL), penicillin (100 units/mL), and streptomycin (100 μg/mL) at 37 °C in a 5% CO_2_ and 95% humidity atmosphere.

### 4.2. MTT Assay

Cell viability was assessed using the MTT assay. In this assay, 1 × 10^4^ cells per well were seeded into sterile 96-well plates and incubated overnight. These cells were treated with increasing concentrations of pardaxin (10 and 25 μg/mL) and incubated for 24 and 48 h, respectively. After incubation, 0.5 μg/μL of MTT was added and cells were incubated for additional 2 h at 37 °C in the dark. Later, the medium was removed and formazan crystals were dissolved in DMSO. Cellular metabolism was determined by measuring the color development at 570 nm using a multiwall scanning spectrophotometer.

### 4.3. Colony Formation

The colony formation ability at a low cell density was determined by plating 1 × 10^3^ cells per well onto a six-well plate and subsequently culturing them for 7 days. Later, SCC-4 cells were stained with 0.5% crystal violet in 30% ethanol and 3% formaldehyde for 10 min at room temperature [33].

### 4.4. Apoptosis

Fixed cells were permeabilized by incubating them in PBS/0.1% Triton X-100 for 5 min at room temperature. The cell slides were washed three times in PBS for 5 min at room temperature. The cell slides were drained and 200 μL of a blocking buffer was added. The slides were laid flat in a humidified chamber and incubated for 1–2 h at room temperature. The caspase-3 antibody (100 μL; 1:200) was added and the slides were subsequently incubated in a humidified chamber overnight at 4 °C. The slides were washed with PBS and then incubated with 100 μL of an FITC-conjugatedsecondary antibody dissolved in PBS (1:500) for 2 hat room temperature. The fluorescence intensity of caspase-3 was measured using a fluorescence microscope.

### 4.5. Cell Cycle

SCC-4 cells were subjected to PI staining for fluorescence-activated cell sorting (FACS) analysis. SCC-4 cells (3 × 10^5^ cells/well) were seeded into sterile six-well plates. After 12-h incubation withpardaxin at various concentrations of 0, 5, 10, 25 μg/mL, the cells were detached using trypsin-EDTA, washed with PBS, collected through centrifugation at 450× *g* for 10 min, and stained with the PI staining solution, containing 50 μg/mL of PI, 0.5% (*w*/*v*) RNase A, and 0.1% (*v*/*v*) Triton-X 100. After incubation for 30 min at 4 °C in the dark, the cell cycle distribution was analyzed using flow cytometry on a FACS Calibur flow cytometer (Becton Dickinson and Company, Mountain View, CA, USA). A total of 100,000 events in each sample were acquired. The cell cycle distribution was determined using the CellQuest Pro software (Becton Dickinson and Company,Franklin Lakes, NJ, USA) [33].

### 4.6. Western Blot Technique

SCC-4 cells were rinsed with ice-cold PBS and lysed in an RIPA lysis buffer with protease and phosphatase inhibitors for 20 min on ice. Later, the cells were centrifuged at 12,000× *g* for 10 min at 4 °C. Protein extracts (20 μg) were resolved through sodium dodecyl sulfate-polyacrylamide gel electrophoresis (200 V, 45 min). Protein bands were electrotransferred to nitrocellulose membranes (80 V, 120 min). Membranes were treated with a 5% enhanced chemiluminescence (ECL) blocking agent (GE Healthcare Bio-Sciences,Little Chalfont, UK) in a saline buffer (T-TBS) containing 0.1% Tween-20, 10 mM Tris-HCl, 150 mM NaCl, 1 mM CaCl_2_, and 1mM MgCl_2_ (pH = 7.4) for 1 h. Later, the membranes were incubated with the primary antibody overnight at 4 °C and subsequently washed three times in T-TBS. The bound antibodies were detected using appropriate horseradish peroxidase-conjugated secondary antibodies and, subsequently, an ECL Plus Western blotting detection system (GE Healthcare Bio-Sciences). ECL was detected using the Molecular Imagers ChemiDoc mod. MP System (Bio-Rad Laboratories) and acquired using ImageLab Software, Version 4.1.

### 4.7. Syrian Hamster Model

In this model, 5–6 week old Syrian (golden) hamsters were procured from the National Laboratory Animal Center (Taipei, Taiwan). The hamsters were housed in polypropylene cages at 27 ± 2 °C and 55 ± 5% humidity with a 12-h light/dark cycle. The hamsters were pretreated by painting the entire mucosal surface of the left buccal pouch with a 0.5% DMBA solution in mineral oil three times a week for 8 weeks. The hamsters in the control group were pretreated with mineral oil only[1,3]. Tumors were collected from the sacrificed hamsters after the 8-week pretreatment and 4-week pardaxin treatment to determine the tumor volume and burden. The tumor volume was calculated using the formula, V = 4/3 (D1/2)(D2/2)(D3/2), where D1, D2, and D3 are three diameter readings (mm) of tumors. The tumor burden was calculated as the product of the total number of tumors and mean volume, as reported in previous studies [1,3].

### 4.8. Assay for PGE2 Level

Mice with DMBA-induced tumors in the buccal pouch were sacrificed to extract the blood. Serum was separated from the clots through centrifugation. Later, serum PGE_2_ levels among the mice in different treatment groups were analyzed using a PGE_2_ Parameter Assay kit (R&D Systems, Minneapolis, MN, USA).

### 4.9. Statistical Analysis

Each experiment was repeated three times. Quantitative data were presented as the mean ± SD. The differences and correlations between the two groups were assessed. A multiple group comparison was conducted using ANOVA with a posttest for subsequent individual group comparisons. All statistical analyses were conducted using SPSS version 13.0 (SPSS, Chicago, IL, USA). Data were considered statistically significant when *p* < 0.05.

## 5. Conclusions

The present study demonstrated a correlation between pardaxin treatment and the serum PGE_2_ level in the DMBA-induced mouse oral cancer model. Pardaxin contributes to OSCC progression and is a potential and promising anticancer agent for oral cancer therapy.
